# Mining GEO and TCGA Database for Immune Microenvironment of Lung Squamous Cell Carcinoma Patients With or Without Chemotherapy

**DOI:** 10.3389/fonc.2022.835225

**Published:** 2022-02-08

**Authors:** Huiping Qiang, Jiaqi Li, Qing Chang, Yinchen Shen, Jialin Qian, Tianqing Chu

**Affiliations:** Department of Respiratory Medicine, Shanghai Chest Hospital, Shanghai Jiao Tong University, Shanghai, China

**Keywords:** lung squamous cell carcinoma, chemotherapy, TCGA, immune microenvironment, tumor mutation burden

## Abstract

**Background:**

Chemotherapy is the main treatment for patients with lung squamous cell carcinoma (LUSC). However, how chemotherapy affects their immune system is rarely reported. This study was aimed to compare the differences in the immune microenvironment of LUSC patients with or without chemotherapy.

**Methods:**

A total of 494 LUSC samples were obtained from The Cancer Genome Atlas (TCGA) database. The immune cell infiltration was evaluated by the ssGSEA algorithm, and the tumor subtype was assayed by ConsensusClusterPlus. The differences in tumor mutation burden (TMB) and clinical information between the two types were then compared. Additionally, the differentially expressed genes (DEGs) between two types were analyzed and hub genes were validated in the GEO database.

**Results:**

LSCC samples in TCGA were divided into three subtypes. Then, combining the tumor subtype and immune scores, the samples were divided into hot and cold tumors. Regardless of whether LUSC patients received chemotherapy, the survival of the hot tumor group was not significantly prolonged compared with that of the cold tumor group. For LUSC patients who received chemotherapy, the TMB value in hot tumor group was significantly higher. Total 501 DEGs were identified between two groups. The high expressions of hub genes *CD19*, *CTLA4*, *FCGR3B*, *CD80*, *IL-10*, etc. were also validated in the GSE37745 dataset.

**Conclusion:**

Chemotherapy does not affect the survival and prognosis of LUSC patients, but it significantly increases the TMB value of patients with hot tumor. The DEGs, especially hub genes, such as *CD19*, *CTLA4*, and *FCGR3B*, may serve as biomarkers to distinguish cold and hot tumors in LUSC.

## Highlights

1. Chemotherapy does not affect the survival of LUSC patients.

2. Chemotherapy significantly increases the TMB value of LUSC patients with hot tumor.

3. *CD47*, *SIRPA*, and other immune checkpoint genes can serve as biomarkers to help identify the immune microenvironment of LUSC patients.

4. *CD19*, *CTLA4*, and other hub genes can serve as biomarkers to help identify the immune microenvironment of LUSC patients.

## Introduction

Lung cancer is one of the most common malignant tumors with a 5-year overall survival rate of 16%–20% (1, 2). Non-small cell lung cancer (NSCLC) accounts for about 80% of all lung cancer types (3), which consist of two main histologic subtypes: lung squamous cell carcinoma (LUSC; accounting for 55% of all NSCLCs) and lung adenocarcinoma (LUAD; accounting for 30%) (4). LUSC is often diagnosed at the advanced stage with poor prognosis and lacks targeted therapies available compared to LUAD.

Presently, chemotherapy remains the standard treatment for LUSC (5). As is known, tumors are the product of a complex interaction between malignant cells and other normal cells from a single initiating cell to a full tumor. Immune cells are normal cell types that are commonly symbiotic with cancer cells (6). In recent years, accumulating evidence has illustrated the correlation between immunotherapy and immune microenvironment (7). The insufficient tumor-infiltrating lymphocytes and low immunogenicity form an immunosuppressive microenvironment which has led to initial resistance to immunotherapy (8). However, how chemotherapy affects IME has not been well explained.

The establishment of The Cancer Genome Atlas (TCGA) database and Gene Expression Omnibus (GEO) database has helped to generate many large-scale cancer genomic datasets and enabled comprehensive bioinformatics analyses (9). Therefore, in this study, we downloaded the gene expression data of LUSC patients who received chemotherapy from the two databases. The immune cell infiltration and immune scores were evaluated to divide the LUSC into cold and hot tumor type. The differences in tumor mutation burden (TMB) and clinical information between the two types were then compared. Additionally, the differentially expressed genes (DEGs) between two types were analyzed to screen key biomarkers.

## Methods

### Data Sources and Preprocessing

The Illumina HiSeq 2000 gene expression data (normalized FPKM expression level data) of lung squamous cell carcinoma (LUSC) were downloaded from TCGA database. There are 550 samples, including 501 tumor samples and 49 normal samples. Among the tumor samples, 494 samples with clinical survival and prognostic information were retained as the training set. Based on the clinical information of these samples, we further divided them into the chemotherapy group and the non-treatment group and performed a subgroup analysis on the TMB and survival of the two groups, respectively. Additionally, the GSE37745 (10–12) dataset, including 196 samples, was downloaded from the NCBI GEO database, which was detected on the GPL570 Affymetrix Human Genome U133 Plus 2.0 Array platform. Among these samples, 66 had clinical survival and prognostic information, which were used as the validation set.

### Analysis of Immune Cell Proportion

Cells in the tumor microenvironment can cluster into different types, and there are robust cell infiltration patterns among these cells. In this study, gene set variation analysis for microarray (GSVA) version 1.36.3 (13) in R3.6.1 based on the single-sample gene set enrichment analysis (ssGSEA) algorithm (14) was used to quantify the infiltration of 28 immune cell types.

### Analysis of Sample Subtypes Based on Immune Cell Proportion

Based on the obtained immune cell proportion, the tumor subtypes were analyzed for all samples using the ConsensusClusterPlus version 1.54.0 (15) in R3.6.1. Based on the disease subtypes, the correlation of survival and prognosis among sample groups of different disease subtypes was evaluated using the Kaplan–Meier (KM) curve method in R3.6.1 survival package version 2.41-1 (16).

### Analysis of Cold Tumor and Hot Tumor Type

ESTIMATE score, immune score, stroma score, and tumor purity were calculated using the estimate package (17) in R3.6.1. Then, hierarchical clustering was performed for the immune cell proportion according to different subtypes using the pheatmap version 1.0.8 (18, 19) in R3.6.1. ESTIMATE score, immune score, stroma score, and tumor purity were presented according to sample distribution. Finally, tumor types were classified into “cold” and “hot” based on ESTIMATE score, immune score, stroma score, and tumor purity, and clinical information of cold and hot tumor was statistically compared using Fisher’s accurate test in R3.6.1.

### TMB Analysis

Based on the mutation information of tumor samples downloaded from TCGA database, the mutation of each gene in each sample was analyzed, and the genes with high-frequency mutation were displayed. Then using the maftools package version 2.6.05 (20) of R3.6.1, the TMB of tumor samples was calculated, and the TMB differences between cold and hot tumor groups were compared.

### Screening of DEGs Associated With Cold and Hot Tumor Groupings

For the tumor samples in TCGA, significant DEGs between hot and cold groups were screened using the limma package version 3.34.7 (21) in R3.6. False discovery rate (FDR) < 0.05 and |log2 fold change (FC)| > 1 were used as the threshold for screening DEGs. Later, DAVID version 6.8 (22) was used for enrichment analysis of the biological process and KEGG signaling pathway for the significant DEGs, and FDR < 0.05 was selected as the threshold.

### Differences in Expression Levels of Immune Checkpoint Gene Between Different Tumor Types

Based on the gene expression level in TCGA samples, the expression levels of specific immune checkpoint genes were extracted, including PD-L1 (*CD274*), PD1 (*PDCD1*), CTLA-4 (*CTLA4*), Tim3 (*HAVCR2*), CD278 (*ICOS*), *LAG3*, *CD47*, *CD73*, *TIGIT*, *BTLA*, myd1 (*SIRPA*), 4-1BB (*TNFRSF9*), OX40 (*TNFRSF*4), and B7-H4 (*VTCN1*). The expression differences between cold and hot tumor groups were compared.

### Construction of Interaction Networks and Screening of Important Genes

The interaction relationship between DEG product proteins was searched from STRING (23) database version 11.0, and interaction scores higher than 0.7 were selected to build the interaction network. The network was visualized through Cytoscape version 3.6.1 (24). Then the network topology was analyzed to screen the hub nodes in the network.

### Validation of Hub Genes

In the validation dataset GSE37745, based on the gene expression level detected in the GSE37745 samples, the proportion of immune cells and the tumor subtype were also analyzed. Additionally, ESTIMATE score, immune score, stroma score, and tumor purity were also calculated as above, and finally, the GSE37745 data set samples were also divided into cold and hot tumor samples based on various indicators. Then, the expression levels of hub genes were extracted from TCGA and GSE37745 datasets respectively. The differences in expression levels of cold and hot tumor samples in the two datasets were investigated.

**Figure 1:**
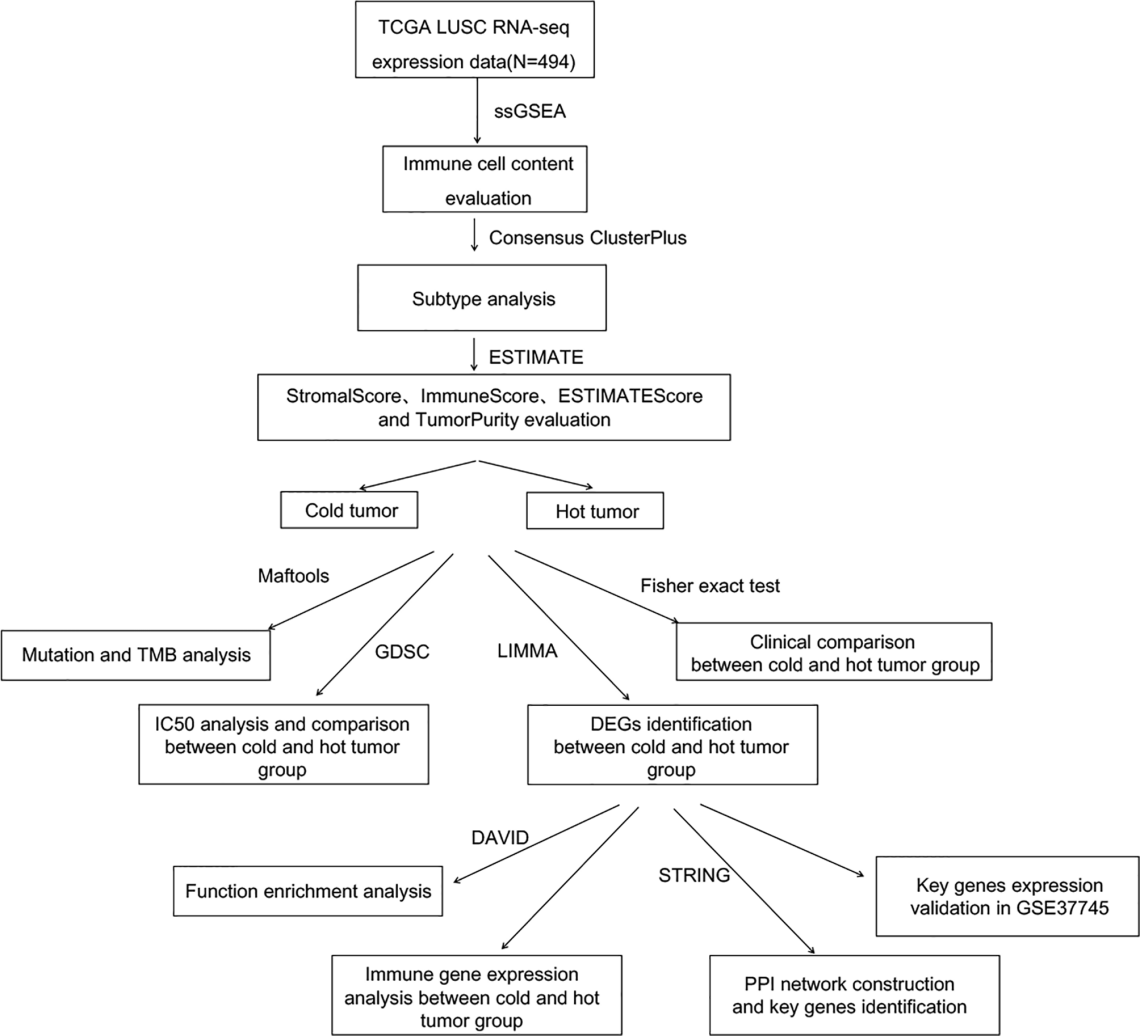
Analysis flowchart.

To systematically describe our study, the analysis flowchart is shown in **Figure 1**.

### Statistical Analysis

R Studio version 3.6.1 and Bioconductor were used for statistical analysis. Overall survival was assessed by KM and log-rank test methods, and subgroup differences were analyzed by the Wilcox test or Kruskal test, with p-values < 0.05 considered to be statistically significant.

## Results

### Proportion of Immune Cells

**Figure 2:**
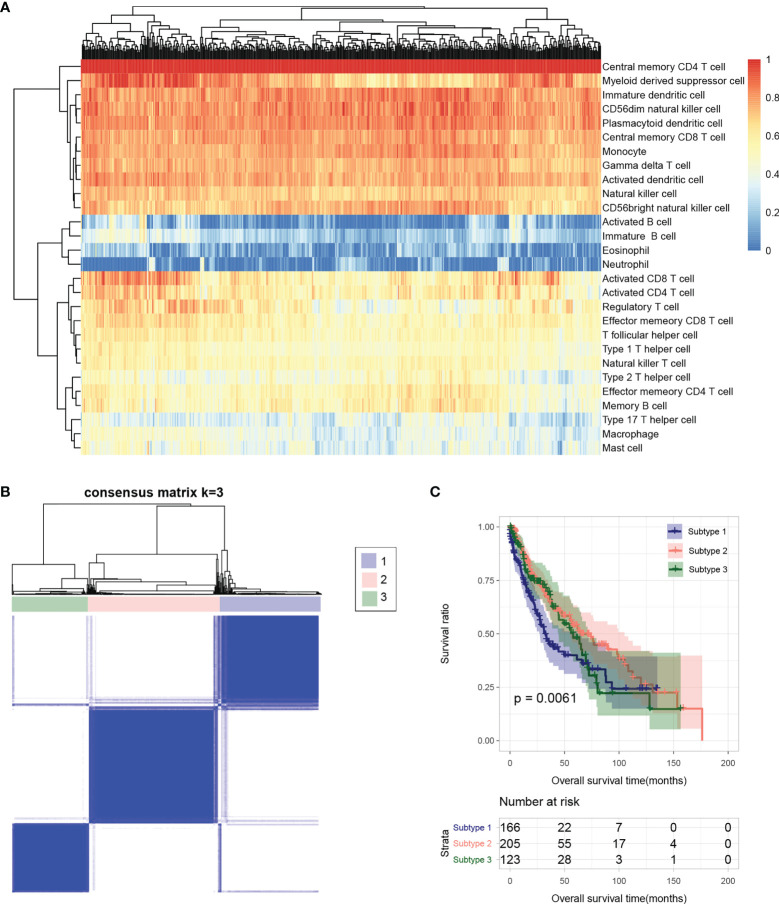
**(A)** Heatmap of sample immune cell proportion evaluated based on ssGSEA. **(B)** Sample subtype analysis cluster diagram. **(C)** KM survival curves of different subtypes.

Based on the gene expression level in tumor samples in TCGA database, the immune cell infiltration of each sample was evaluated. The relative abundance of 28 infiltrating immune cell populations was visualized through a heatmap (**Figure 2A**). Except for activated B cell, immature B cell, eosinophil, neutrophil, type 17 T helper cell, macrophage, and mast cell, the other 21 infiltrating immune cell populations had a higher proportion in almost all samples.

### Tumor Subtype Analysis

The flow chart of bioinformatic analysis is shown in **Figure 1**. Based on the identified immune cell proportion in samples, subtypes were analyzed for tumor samples. As shown in **Figure 2B**, three subtypes were obtained, and there were 166, 205, and 123 tumor samples in subtype 1, 2, and 3, respectively. KM survival analysis showed that there were significant differences in survival and prognosis information among different subtypes, among which subtype 2 samples had better clinical prognosis (**Figure 2C**).

### Cold and Hot Tumor Typing

**Figure 3:**
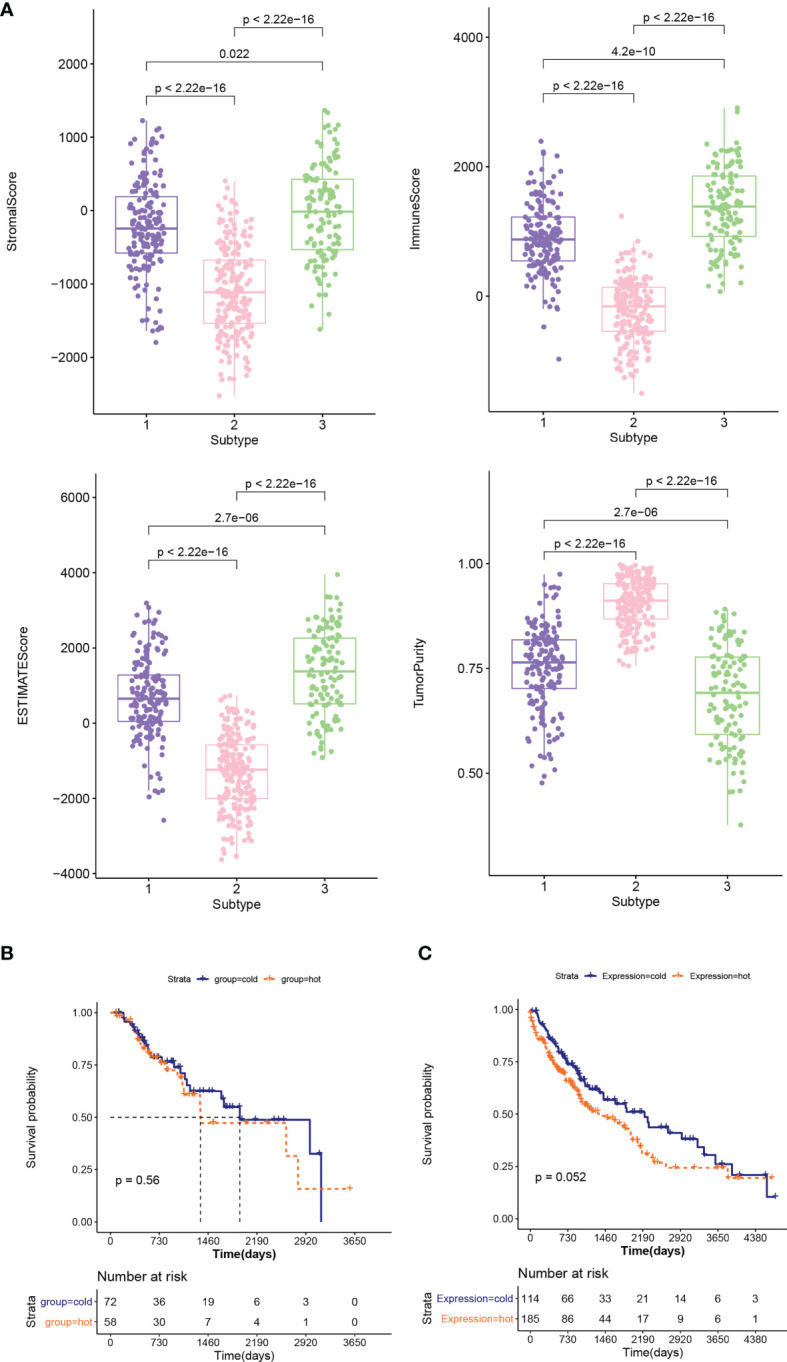
**(A)** Comparison of the distribution of stroma score, ESTIMATE score, immune score, and tumor purity in different subtypes. **(B, C)** KM curves associated with survival outcomes between cold and hot tumor groups in chemotherapy **(B)** and non-treatment groups **(C)**.

The distribution characteristics of ESTIMATE score, immune score, stroma score, and tumor purity in different subtypes were compared, as shown in **Figure 3A**. The distribution of each score in different subtypes was significantly different. The distributions of ESTIMATE score, immune score, and stroma score were the lowest in subtype 2 and higher in subtype 1 and 3. However, the distribution trend for tumor purity was reversed. Subsequently, the proportion of immune cells was hierarchical clustering according to different subtype groups, and ESTIMATE score, immune score, stroma score, and tumor purity were also presented according to sample distribution. Then, according to reference (23), combined with ESTIMATE score, immune score, stroma score, and tumor purity information, we defined subtype 2 as “cold” type and combined subtypes 1 and 3 as “hot” type. After a subgroup analysis of patients based on whether they received chemotherapy, there was no significant difference in the clinical prognosis between cold and hot tumor groups in both chemotherapy (**Figure 3B**) and non-treatment groups (**Figure 3C**).

### TMB Analysis

**Figure 4:**
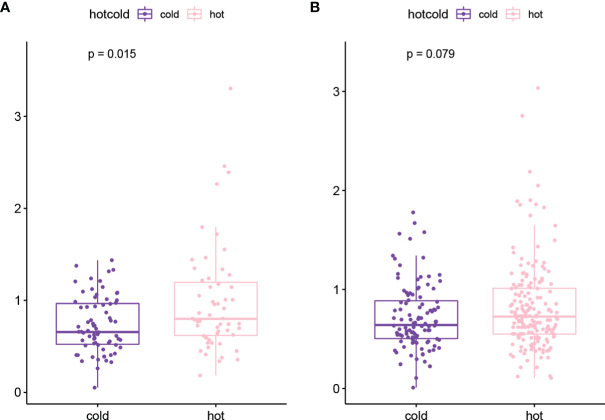
The TMB value of hot tumor group and cold tumor group in chemotherapy **(A)** and non-treatment **(B)** groups.

The genes with high-frequency mutations are shown in **Figure S1A**, including 20 genes, such as tumor protein P53 (*TP53*), titin (*TTN*), and CUB and sushi multiple domains 3 (*CSMD3*). Then TMB values of tumor samples were calculated. The results showed that the TMB value in the hot tumor group was significantly higher than that in the cold tumor group (**Figure S1B**). In the stratified analysis, the results of the chemotherapy group were consistent with the overall result. The TMB value of the hot tumor group was higher than that of the cold tumor group (**Figure 4A**), while in the non-treatment group, no significant difference was found in the TMB value between two groups (**Figure 4B**).

### DEGs Between Cold and Hot Tumor Groups

**Figure 5:**
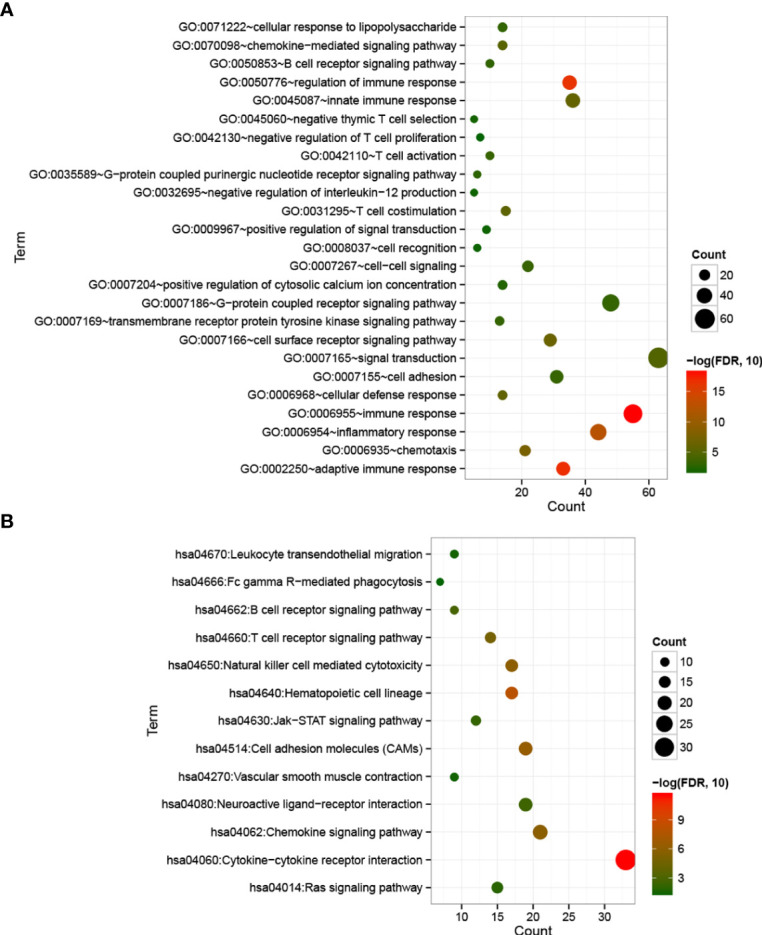
Bubble maps of biological processes **(A)** and KEGG signaling pathways **(B)** associated with significantly differentially expressed genes. The horizontal axis represents the number of differentially expressed genes, the vertical axis represents the name of the item, and the size of the dot represents the number of DEGs.

With FDR < 0.05 and |log2 fold change (FC)| > 1, 501 DEGs were identified between cold and hot tumor groups, which were significantly correlated with 25 biological processes and 13 KEGG signaling pathways. As shown in **Figure 5A**, these biological processes were mainly associated with immune response and inflammatory response. The top five pathways with lower FDRs were cytokine–cytokine receptor interaction, hematopoietic cell lineage, cell adhesion molecules (CAMs), natural killer cell-mediated cytotoxicity, and chemokine signaling pathway (**Figure 5B**).

### Expression Level of Specific Immune Checkpoint Genes in Cold and Hot Tumor Groups

**Figure 6:**
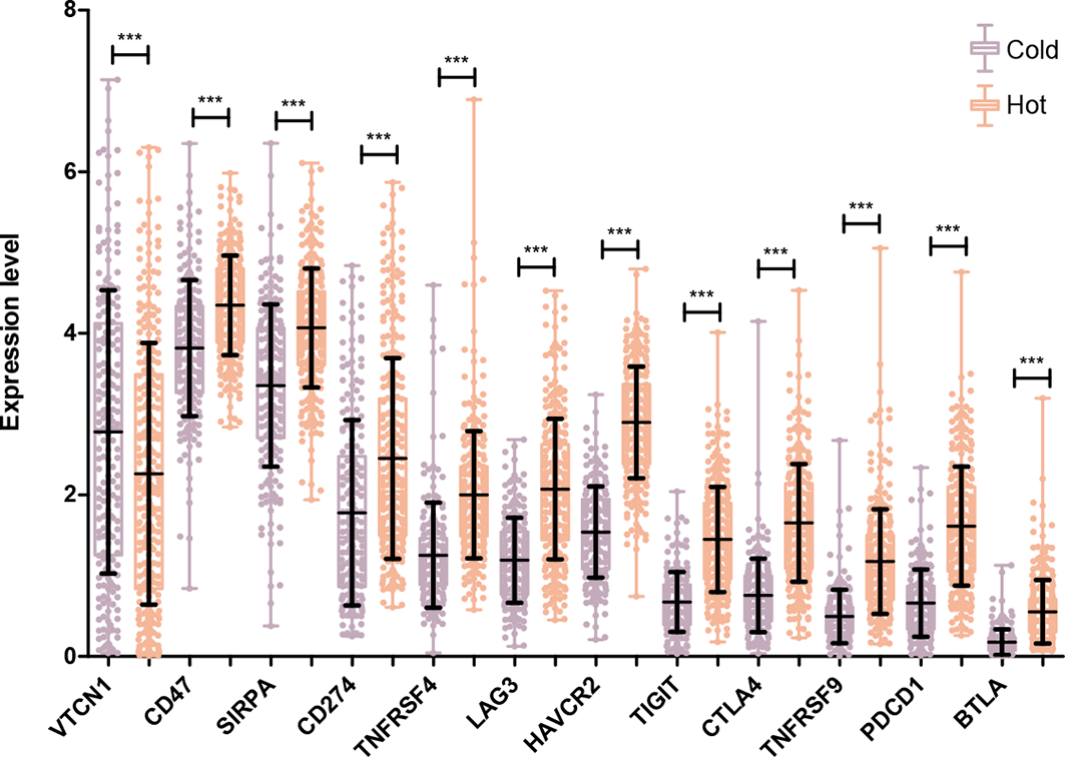
Comparison of specific immune checkpoint genes expression between Hot and Cold groups. ***P < 0.001.

The expression levels of 14 immune checkpoint genes were extracted from the LSCC samples in TCGA database. As shown in **Figure 6**, there was no expression information for *ICOS* and *CD73*. For the other 12 genes, their expression levels in the hot tumor group were significantly higher than that in the cold tumor group, except for *VTCN1*.

### Construction of Interaction Networks and Screening of Hub Genes

**Figure 7:**
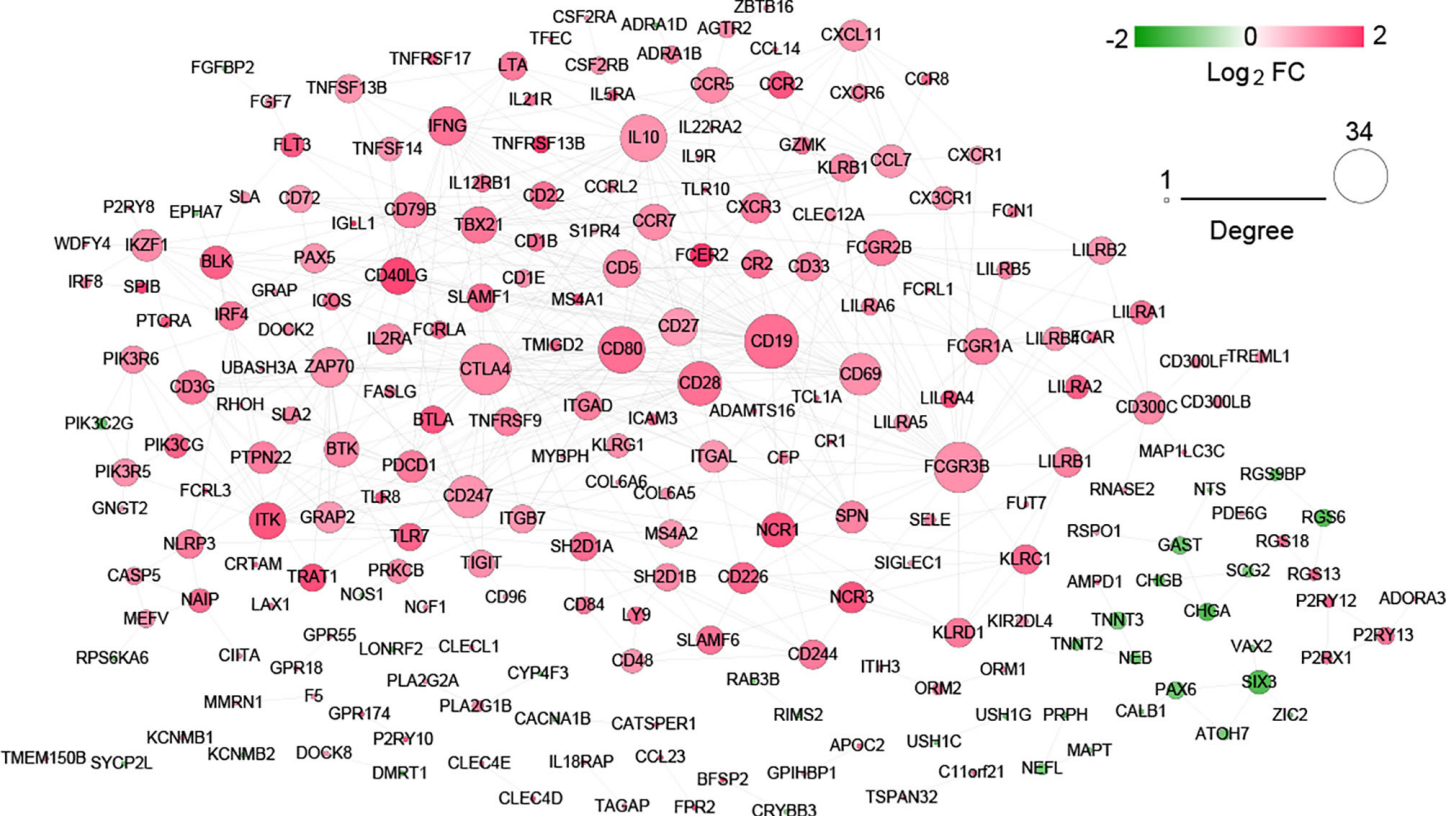
Protein interaction network of significantly differentially expressed genes.

After searching in the STRING database, 517 interaction pairs with interaction scores more than 0.7 were obtained, and a network with 219 nodes and 517 edges was established (**Figure 7**). Following network topology analysis, the top10 genes were selected as hub genes in the network according to the rank of node connectivity from large to small. The top10 genes were CD19, cytotoxic T-lymphocyte antigen 4 (CTLA4*)*, Fc fragment of IgG receptor IIIb (FCGR3B), CD80, interleukin 10 (IL10), CD28, CD247, CD69, zeta chain of T cell receptor-associated protein kinase 70 (ZAP70), and interferon gamma (IFNG) (**Table S1**).

### Validation of Hub Genes

**Figure 8:**
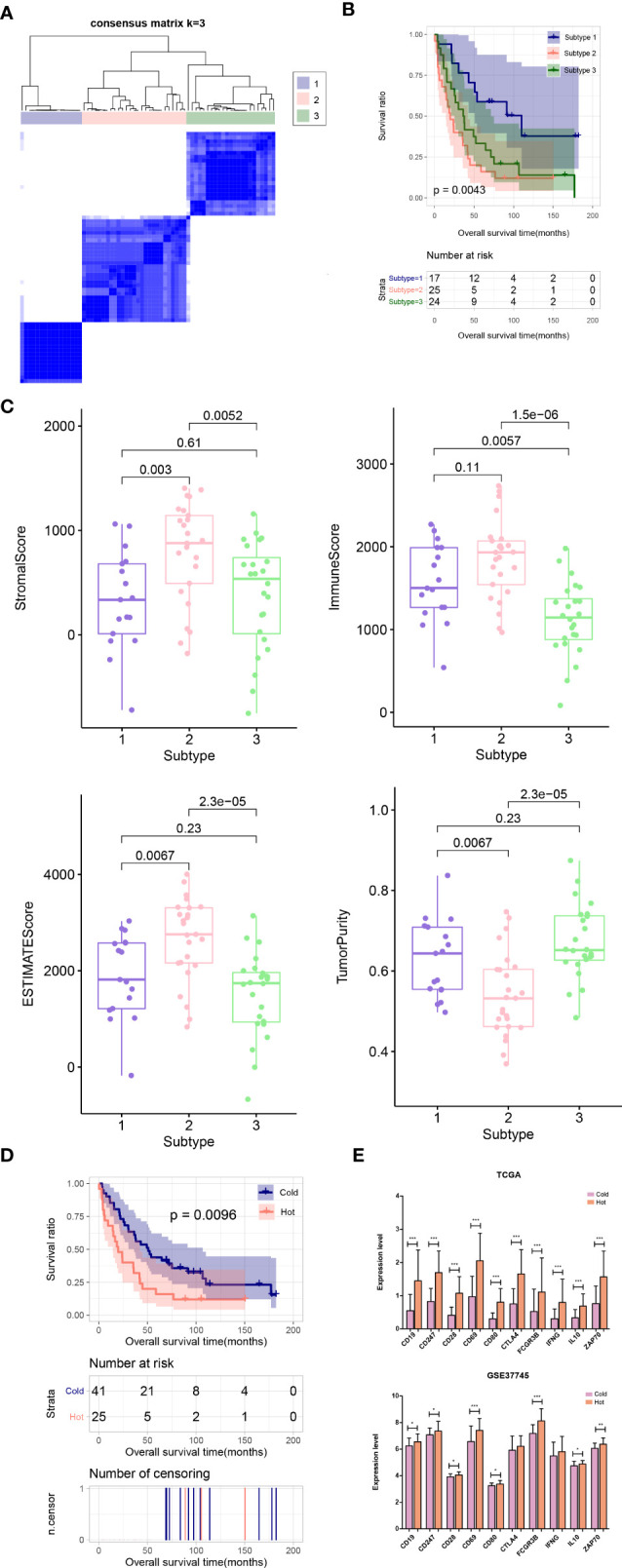
**(A)** Sample subtype analysis cluster diagram in the validation set. **(B)** KM survival curves of different subtypes in validation set. **(C)** Comparison of the distribution of stroma score, ESTIMATE score, immune score, and tumor purity in different subtypes in validation set. **(D)** KM curves associated with survival outcomes in the Hot and Cold sample groups in validation set. **(E)** Expression level distribution of top10 hub genes in Cold and Hot tumor samples in the TCGA training set and validation dataset GSE37745. *P < 0.05; **P < 0.01; ***P < 0.001.

In the validation dataset GSE37745, the proportion of immune cells was evaluated, and then the subtype of the samples was analyzed based on the immune cell proportion. The samples were divided into 3 subtypes. Subtypes I, II, and III contained 17, 25, and 24 tumor samples, respectively (**Figure 8A**). The subtype II group had poor clinical survival prognosis, and subtypes I and III had better clinical prognosis (**Figure 8B**). The comparison results of ESTIMATE score, immune score, stroma score, and tumor purity were similar to that in the training set (**Figure 8C**). Moreover, according to the grouping rules for hot and cold tumor types in the training set, the validation dataset samples were also divided into hot and cold tumor. There were significant differences in clinical prognosis between the two types of tumor samples, and the cold tumor group had good survival prognosis (**Figure 8D**), which was consistent with the results in the training set.

Furthermore, the expression levels of the hub genes were extracted from TCGA and GSE37745 datasets. All of the ten genes were significantly upregulated in the hot tumor group compared with the cold group in the TCGA dataset. In GSE37745, except for CTLA4 and IFNG, the other genes were also significantly upregulated in hot tumor group (**Figure 8E**).

## Discussion

In recent years, the treatment of lung cancer has become more and more diverse. However, compared with LUAD, LUSC lacks driver gene mutations and standard chemotherapy is still the main treatment option. TCGA has revealed the genomic data from a large number of tumor samples and has provided detailed information about the tumor immune microenvironment (25, 26). Immune heterogeneity in the tumor microenvironment is associated with prognosis and drug sensitivity of patients with many types of cancers (27). It has been suggested that low immune cell infiltration is linked with poor clinical outcomes for patients with cancer. Analysis of immune signatures may reveal biomarkers for clinical outcome assessment (28). In order to explore whether LUSC patients receiving chemotherapy has an impact on the baseline immune microenvironment, we conducted this study. The LUSC samples downloaded from TCGA database were divided into three subtypes based on the immune cell proportion. Subtype 2 had the lowest ESTIMATE, stroma, and immune scores. In accordance with the report above, the samples of subtype 2 had the best clinical prognosis. Preclinical studies demonstrate that the majority of chemotherapeutic drugs exert immunostimulatory effects, either by inhibiting immunosuppressive cells and/or activating effector cells, or by increasing immunogenicity and increasing T-cell infiltration (29), whereas for LUSC patients with different degrees of immune infiltration in our study, chemotherapy did not significantly prolong their survival. There possible reasons are as follows: on the one hand, the sample size of patients receiving chemotherapy was small, and the results were not representative; on the other hand, myelosuppression and leukocytopenia caused by chemotherapy may affect the survival of LUSC patients.

TMB refers to the number of somatic mutations per 1 million bases, excluding single-nucleotide polymorphism (SNP), germline, copy number variation, and structural variation (30). TMB is an emerging characteristic of cancer and is associated with microsatellite instability. Highly mutated tumors may contain neoantigens, making them susceptible to immune cells (31). The increase of TMB in the human cancer genome is attributed to endogenous factors and environmental damage. Previous studies reported that patients with high TMB have a significantly better response to immunotherapy (32). Thus, TMB has become a biomarker for predicting the efficacy of immunotherapy. In our study, the TMB values between two groups were analyzed and we found that TMB in the cold group was significantly lower than that of the hot group. Furthermore, in all LUSC patients receiving chemotherapy, the TMB value of the hot tumor group was also obviously higher than that of the cold tumor group. These results demonstrated that high TMB often has a relatively favorable living condition. Based on this, we speculated that the hot tumor group that had a higher TMB may be more susceptible to immune checkpoint inhibitors after first-line chemotherapy advancement in LUSC. The correlation between TMB and tumor-infiltrating immune cells was analyzed to reflect on the status of the immune microenvironment.

A total of 501 DEGs were identified between two groups, which were enriched in immune response-related functions. In the present study, 12 immune checkpoint genes were found to be significantly differentially expressed between hot and cold tumor groups. Among those immune checkpoint genes, both CD47 and SIRPA were remarkably upregulated in the hot tumor group. CD47 is an integrin-associated protein and is overexpressed in many cancer cells (33). SIRPA is a main receptor of CD47 (34). In some human cancers, CD47 binds to SIRPA to trigger the inhibitory signaling pathway that caused tumor cells to evade from phagocytosis by macrophages (35). Now, tumor immunotherapy targeting the CD47/SIRPA axis has also become a hotspot in cancer treatment (36). We speculated that these immune checkpoint genes may be biomarkers for immunotherapy after chemotherapy in LUSC patients with more immune infiltration.

Moreover, ten hub genes, such as CD19, CTLA4, FCGR3B, and CD80, were validated in the GEO database. Interestingly, these genes were all upregulated in the hot tumor group. Additionally, among the ten genes, five were CD molecular, such as CD19, CD80, and CD28. CD19 is a transmembrane glycoprotein of the immunoglobulin superfamily and is broadly expressed in B-cell malignancies. CD80 can be expressed in immune cells as well as some cancer cells. Moreover, it could interact with both coinhibitory (CTLA4) and costimulatory (CD28) receptors to regulate the immune response (37). Specially, CTLA4 is also an immune checkpoint gene in the present study. CTLA4 is considered as an inhibitory regulator of T-cell activation. Blockading the physiological function of CTLA4 in T cells is now used as a therapeutic approach in many human malignancies, including NSCLC (38). FCGR3B encodes the activator Fc receptor, which functions in the regulation of immune and inflammatory responses (39). Its role in LUSC immunotherapy has not been reported to our knowledge. Given its role in immune and inflammatory responses, we speculated that FCGR3B may serve as a biomarker to distinguish cold and hot tumors in LUSC. For instance, the results of the current study were not validated using an independent patient cohort. Thus, further *in vitro* or *in vivo* experiments are needed to validate our findings.

## Conclusion

In conclusion, chemotherapy does not affect the survival of LUSC patients in our study, but it significantly increases the TMB value, suggesting that subsequent immunotherapy may further improve the efficacy and improve the prognosis. *CD47*, *SIRPA*, and other immune checkpoint genes, as well as *CD19*, *CTLA4*, and other hub genes, can serve as biomarkers to help identify the immune microenvironment of LUSC patients, so as to better screen people who are suitable for continuing immunotherapy.

## Data Availability Statement

The original contributions presented in the study are included in the article/**Supplementary Material**. Further inquiries can be directed to the corresponding authors.

## Author Contributions

HQ and JL contributed to the conception/design of the work, the collection and analysis of data, and the writing and edit of the article. The remaining authors provided editing and writing assistance. All authors contributed to the article and approved the submitted version.

## Funding

This study was funded by the Western Medicine Guide Project of Shanghai Committee of Science and Technology (grant no. 21Y11913500).

## Conflict of Interest

The authors declare that the research was conducted in the absence of any commercial or financial relationships that could be construed as a potential conflict of interest.

## Publisher’s Note

All claims expressed in this article are solely those of the authors and do not necessarily represent those of their affiliated organizations, or those of the publisher, the editors and the reviewers. Any product that may be evaluated in this article, or claim that may be made by its manufacturer, is not guaranteed or endorsed by the publisher.

## Supplementary Material

Supplementary Figure 1
**(A)** Genes with high-frequency mutations in LUSC patients; **(B)** TMB value in the hot tumor group and cold tumor group.
