# Supplementary figures and images for: Mining GEO and TCGA Database for Immune Microenvironment of Lung Squamous Cell Carcinoma Patients With or Without Chemotherapy
Source: Front Oncol. 2022 Feb 8;12:835225. doi: 10.3389/fonc.2022.835225 (PMC8861363; doi:10.3389/fonc.2022.835225)

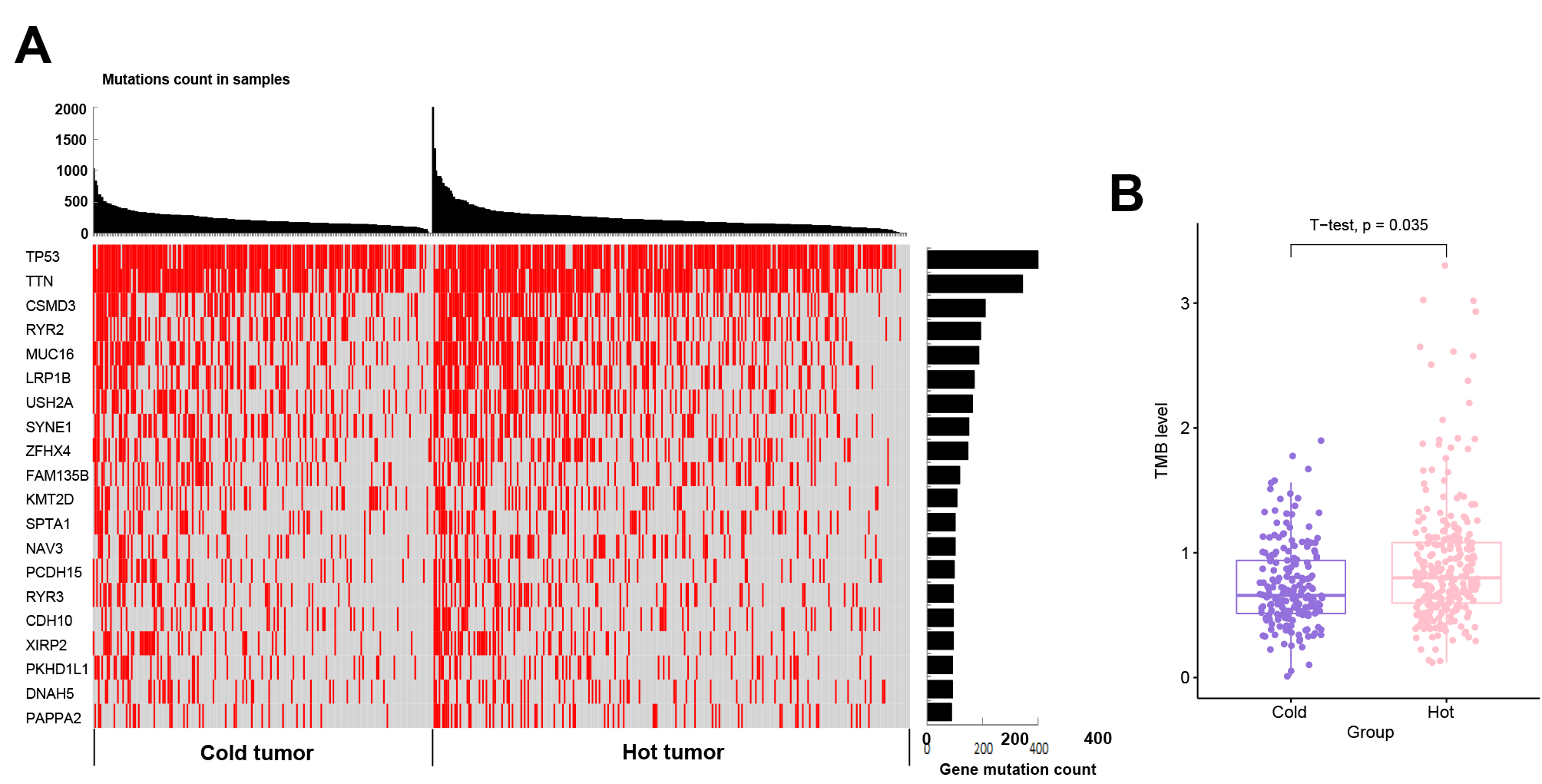

Supplement: Supplementary Figure 1 — (A) Genes with high-frequency mutations in LUSC patients; (B) TMB value in the hot tumor group and cold tumor group. [file Image_1.tif]
