# Supplementary material for: Mining GEO and TCGA Database for Immune Microenvironment of Lung Squamous Cell Carcinoma Patients With or Without Chemotherapy
Source: Front Oncol. 2022 Feb 8;12:835225. doi: 10.3389/fonc.2022.835225 (PMC8861363; doi:10.3389/fonc.2022.835225)
Supplement: Supplementary file 2 [file Table_1.doc]

**Table S1** Hub genes in the protein-protein interaction network

| **Node** | **AverageShortestPathLength** | **Betweenness** | **Centrality** | **Degree** |
| --- | --- | --- | --- | --- |
| CD19 | 2.20988 | 0.23984 | 0.45251 | 34 |
| CTLA4 | 2.26543 | 0.11190 | 0.44142 | 31 |
| FCGR3B | 2.36420 | 0.17253 | 0.42298 | 30 |
| CD80 | 2.34568 | 0.06536 | 0.42632 | 26 |
| IL10 | 2.33951 | 0.15433 | 0.42744 | 26 |
| CD28 | 2.36420 | 0.04059 | 0.42298 | 23 |
| CD247 | 2.44444 | 0.05858 | 0.40909 | 21 |
| CD69 | 2.41358 | 0.03807 | 0.41432 | 21 |
| ZAP70 | 2.69136 | 0.05159 | 0.37156 | 18 |
| IFNG | 2.66667 | 0.01444 | 0.37500 | 17 |
